# Upstream or swim up processing technique: which one is more effective to select human sperm with high chromatin integrity

**Published:** 2018-07

**Authors:** Mahnaz Heidari, Niknam Lakpour, Mahsa Darbandi, Sara Darbani, Saeideh Shani, Leila Goharbakhsh, Ghazaleh Cheshmi, Mohammad Mehdi Akhondi, Mohammad Reza Sadeghi

**Affiliations:** 1 *Department of Embryology and Andrology, Reproductive Biotechnology Research Center, Avicenna Research Institute, ACECR, Tehran, Iran.*; 2 *Department of Embryology and Andrology, Avicenna Infertility Clinic, Tehran, Iran.*; 3 *Department of Monoclonal Antibody Research Center, Avicenna Research Institute (ARI), ACECR, Tehran, Iran.*

**Keywords:** Chromatin maturation, DNA fragmentation, Sperm, Swim up, Upstream technique

## Abstract

**Background:**

Sperm processing methods separate motile sperms with good morphology from dead and abnormal forms of sperms, immature germ cells, and non-sperm cells.

**Objective:**

The propose of this study was to compare the efficacy of upstream and swim-up processing techniques to separate sperms with the high quality especially in relation to sperm chromatin integrity.

**Materials and Methods:**

This experimental study used semen samples from 60 normozoospermic men. Specimens were divided into equal aliquots for processing by swim up (group A), and upstream (group B) methods and compare with control by raw semen (group C). Sperm concentration, morphology, motility, DNA fragmentation and chromatin maturation were measured in these three groups.

**Results:**

The results revealed that sperm concentration in the swim up samples was significantly greater than upstream samples (p≤0.04). as addition, motile sperm recovery including the percentage of progressive motility and a total number of motile sperm was better in the swim-up compared to an upstream method and raw semen (p≤0.001). The cell debris and seminal fluid were equally removed by both methods and the percentage of normal forms was also similar in both procedures (p≥0.4). In addition, sperm DNA fragmentation and chromatin maturation were not significantly different between the three groups (p≥0.1).

**Conclusion:**

According to results, apparently the upstream method had no significant efficiency to separate good quality sperms compare to swim up. Therefore, swim up seems to be a simple, inexpensive, reliable and widely available method with an efficient yield to separate motile sperm with good morphology and better chromatin integrity for insemination in the infertility clinics.

## Introduction

Infertility is a most problem n 15-20% of couples in the reproductive ages and Male infertility is Couse of about 50% of all infertile couples of all couples who refer to infertility clinics (1). About 45% of infertile causes is related to male factor so need to use assisted reproductive techniques (ART) (2). ARTs such as intrauterine insemination (IUI), in vitro fertilization (IVF) and intracytoplasmic sperm injection (ICSI), has been commonly useful in this treatment (3). To improve the function of sperm in this process, it is needed to separate high-quality spermatozoa from seminal plasma (4). A separation technique is based on different principles like migration, filtration or density gradient centrifugation (5, 6). 

These processing methods aim to produce sperm suspensions free of seminal plasma, immotile sperm, cell debris, leukocytes, and other contaminants such as bacteria, with a high recovery of motile sperm for use in conventional IVF and ICSI (7). DNA damage may occur during sperm chromatin compaction process which can negatively influence the sperm fertilization ability, so it is important to separate sperms with high genetics condition (8). However, single or multiple step centrifugation damages sperm via the reactive oxygen species (ROS) (9). The suitable sperm preparation technique should also minimize the sperm DNA, protein and lipid damages by in vitro generated ROS (10). Recently it is suggested that sperm separation methods like the swim up and upstream yield a higher number of motile spermatozoa in male cases with oligoasthenoteratospermia. Todays, the conventional swim-up procedure is the most popular, simple and cheapest technique and sperm separation by this method has become a routine technique in many ART laboratories (11). 

It can be done easily and quickly to the recover of a high percentage of motile sperms. Following the development of the classical swim-up method, more complicated techniques were developed to increase the number of motile and normal form of sperm even in severe male factor cases. Theoretically, it seems that because of centrifugation stage omission in the upstream method, using this method may cause less physical damages compare to swim up method (8, 12). 

To facilitate sperm preparation in ART clinics, this study evaluated the upstream method for processing of semen and separation of motile sperm with normal morphology and good DNA compaction compare to swim up a method.

## Materials and methods

**Samples collection**

**Figure 1:**
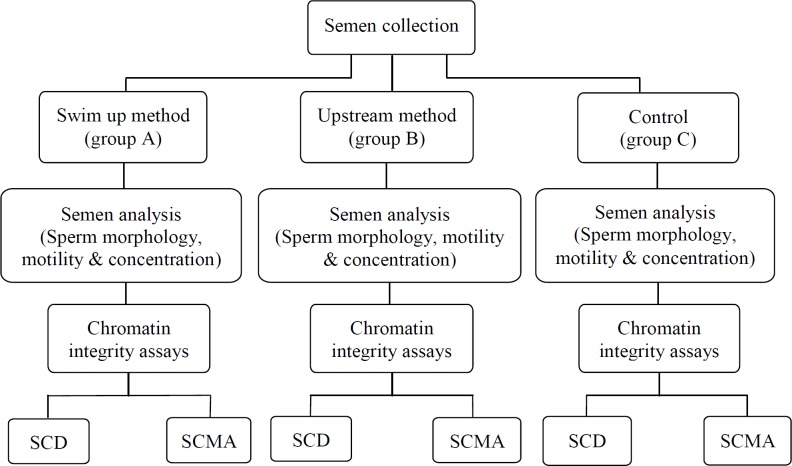
The study design flowchart. Semen collection and processing have been shown in this image

The semen samples were obtained from 60 normozoospermic men who attended the Avicenna Infertility Clinic, Tehran, Iran, for treatment of infertility. Semen specimen was collected within a 2-3 day abstinence period. Semen specimens were produced by masturbation into a sterile container, the remnant of each sample was used for this study following semen analysis (Figure 1). 

**Sperm preparation techniques**

The semen sample was aliquoted into two equal parts after semen analysis and processing was performed according to the following methods:

**Swim up method (group A)**

In standard swim-up technique, following liquefaction, 1 ml of whole semen was gently mixed with 1 ml of Ham’s F10 medium, supplemented with human serum albumin (3%) and centrifuged (330×g for 10 min). The supernatant was removed and 2 ml of “Ham’s F-10” media was added to the pellet. The sperm pellet was used for swim-up method (5-30 min). The sperm concentration, motility, viability, morphology, DNA integrity, and sperm chromatin assay were analyzed-.

**Upstream method (group B)**

This method was performed using the upstream device (Figure II) (Tech Win Co, Iran), according to the instruction of the kit. Semen was deposited on the nylon mesh in upper chamber of the device; supplemented “Ham’s F-10” media was gently added to the top of semen. The device was incubated in 37^o^C for 30 min as seminal plasma going down through nylon mesh, the motile sperm swim up the upper medium. Subsequently, the media contained the motile sperm in the upper chamber was transferred to a sterile tube for further analysis of sperm concentration, motility, viability, morphology, DNA integrity and chromatin maturity.

**Semen with no preparation technique** **(group C)**

Raw semen with no preparation technique was used as a control group.

**Semen analysis**

The semen analysis was performed and normospermic men were selected based on sperm morphology ≥4%, progressive motility ≥32% and concentration ≥20×10^6^ sperm/mL according to WHO guideline(13).

**Sperm chromatin dispersion (SCD)**

This test was performed using the SDFA kit (Dain bioassay Co, Iran), according to the instruction of the kit. 50 μL semen was diluted in Hams F10 and semen aliquot was mixed with agarose (6.5%) and 20 μL of the mixture was loaded onto a pretreated glass slide. The slides were placed on to a cold surface for 5 min at 4°C. 

The slides were treated with a denaturing solution for 7 min and then slides were treated with a lysing solution for 15 min. Following this step, the slides were washed with distilled water for 5 min. slides dehydration were perform using increasing concentrations of ethanol (70%, 90% and 100%, 2 min for each concentration). Finally, air-dried slide was stained. A least 200 sperm were assessed under 1000× magnification of the microscope. Sperm with large or medium halo were classified as intact chromatin and those with no halo or small halo were classified as sperm with fragmented DNA. The result was presented with the percentage of total DNA fragmented sperm (14).

**Sperm chromatin maturation**

Sperm chromatin maturity was assessed according to the instruction of the SCMA kit using the sperm chromatin maturation assay kit (Dain Bioassay Co., Iran). Briefly, at first 1×10^6^ sperm/mL of each sample as centrifuged (300 g, 5 min). Thin smears were prepared by 10 μL of sperm suspension. The slides were air-dried and fixed for 30 min at room temperature with glutaraldehyde. The slides were stained through several steps of staining with aniline blue/ eosin and washed. At least 200 sperm were evaluated in different areas of each slide with a 1000× magnification of the microscope. The pink and the blue sperms were classified as mature and immature sperms respectively. The result was presented with the percentage of total chromatin immature sperm.

**Ethical consideration**

This study was approved by the Ethics Committee of the Avicenna Research Institute, ACECR, Tehran, Iran (93/4886.).

**Statistical analysis**

Data were checked for normality test. Basic descriptive statistics (means±standard deviation) were calculated for different parameters such as sperm concentration, total motility, rapid progressive motility, normal morphology, DNA fragmentation and chromatin immaturity using statistics package for social sciences (SPSS) (version 19, SPSS Inc., USA). ANOVA test was used to compare the differences of variables in the three groups. p<0.05 was considered statistically significant.

## Results

A total of sixty participants fulﬁlled the inclusion criteria of study and each sample was divided into three groups of A (swim-up processing), B (upstream processing) and C (control group without any processing). Sperm concentration, normal morphology, progressive and non-progressive motility, DNA fragmentation and chromatin maturation were assessed for all samples. Group A demonstrated a significant reduction in DNA fragmentation when compared to group B (p=0.06) and C (p=0.004). The means±SD of DNA fragmentation were 22.94±7.85, 24.01±8.53 and 27.83±10.30 in group A, B and C respectively. In addition, sperm normal morphology showed a significant increase in group A compared to group B (p=0.5) and C (p=0.001); 9.05±1.96, 8.69±2.01 and 6.66±2.4 respectively. 

Sperm progressive motility demonstrated a significant increase in group A compared to C (47.59±14.76 vs. 42.07±10.42) (p=0.001). The result showed that although sperm concentration decrease in group A compared to the group C, the concentration of normal sperm in group A was more than group B (p=0.04). The concentration of normal sperm was 40.88±19.15, 33.68±12.02 and 54.76±24.6 in group A, B and C respectively. Also group B showed significant reduction in DNA fragmentation (p=0.02), non-progressive motility (p=0.03) and sperm concentration (p=0.000) compare to group C (Figure 3).

SCMA: Sperm chromatin maturation assay, SCD: sperm DNA chromatin dispersion.

**Figure 2:**
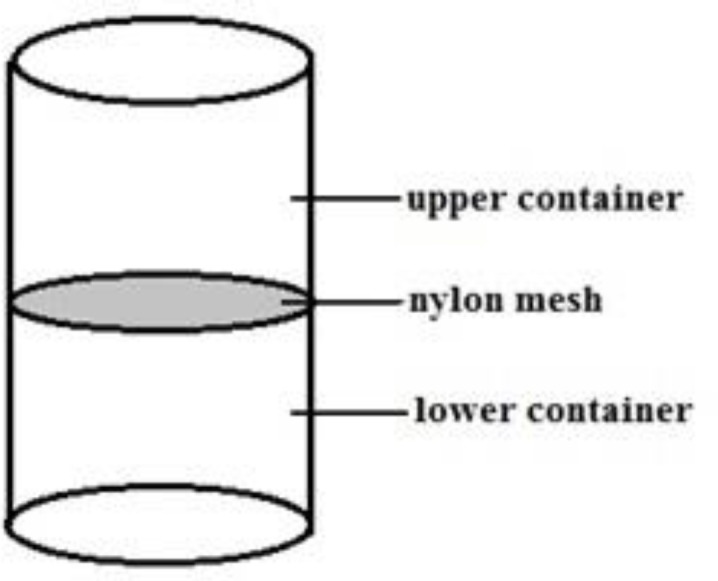
Side view of upstream device. Semen deposited on the nylon mesh in the upper chamber of the device and incubated at 37°C for 30 min as seminal plasma going down through nylon mesh, the motile sperm swims up the upper medium. Subsequently, the media contain the motile sperm in the upper chamber

**Figure 3:**
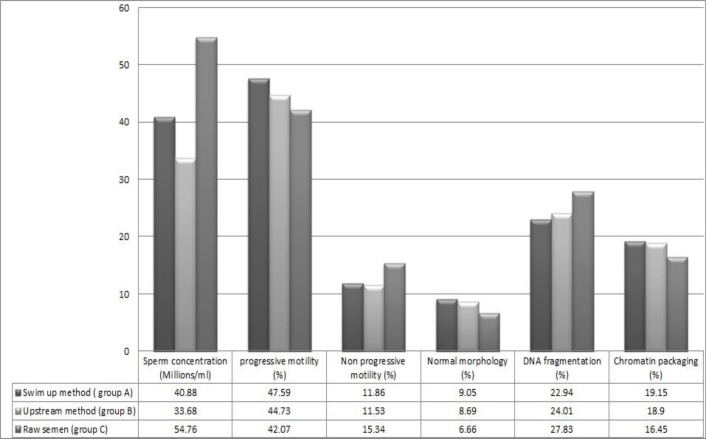
Comparison of sperm parameters between swim up and upstream methods and raw semen (n=60).

## Discussion

Selecting the best sperm is the critical stage of successful ART treatment procedures in infertility clinics. According to our findings swim-up method could signiﬁcantly increase sperm with appropriate concentration, normal morphology, and progressive motility and DNA integrity. Interestingly, the upstream method had a significant increase in sperm concentration, normal morphology, and non-progressive motility after comparison with the control. Likewise, progressive motility and concentration were signiﬁcantly differen in comparison of two preparation methods; it seems that both swim up and upstream methods were beneficial methods to separate high-quality sperm for ART. In addition, we found that sperm prepared with swim-up method in comparison to upstream had more concentration and progressive motility and lower DNA damages. The semen debris was equally removed by both methods and the percentage of normal sperm was also similar in both procedures. Application of sperm processing methods has increased with the development of ARTs. They have different efficacy regarding recovery rates of concentration, motility, morphology, and sperm DNA integrity (15). 

The main aim of sperm processing methods for ARTs is to select the best quality motile sperm from the ejaculate (16). The motility of the sperm in the final processed sample is important for the fertilization rate of human sperm. The best sperm processing method should be mild and provides a highly functional sperm (17). Because the ROS production in spermatozoa and leucocyte cells, It is believed that the semen centrifugation can make sperm dysfunction (18). So, less invasive sperm processing methods such as swim-up procedures and double density gradient centrifugation are commonly useful in clinical practice (19). Formerly, it has been reported that the swim-up semen processing technique can a decrease the sperm DNA fragmentation values. which is a strong predictor of successful pregnancy in ART (20). 

Although, in this study, there was no significant difference between DNA fragmentation and sperm chromatin maturation using swim up and upstream methods. It has been shown that swim up and upstream methods can be most successful for a patient with a normal semen analysis, but it is not recommended for samples of high viscosity and leukcytospermic samples, or samples with a high content of cell debris. The swim up method recovers more motile sperm and routinely used to prepare normal semen. It could obtain and recover sufficiently motile sperm with superior motility in relation to the upstream device. Our findings are similar to a study by Abed *et al* who compared the efficacy of swim-up and upstream methods (17). 

The present study suggests that swim-up and upstream techniques are simple, fast, accurate, and highly reproducible sperm processing methods in ART. Also, we have demonstrated that the swim-up and upstream method is effective in eliminating sperm with fragmented DNA. This study confirms that swim up and upstream methods act as a natural selection mechanism to separate normal motile sperm. However, these techniques lead to the concentration of immature germ cell, leukocyte, dead sperm, and debris. Our observation did not show any significant difference in swim-up technique versus upstream method in the elimination of sperm with damaged DNA. This is in agreement with the result of a study which reported the swim-up technique is unsuccessful to isolate sperm with high chromatin integrity (20). 

Formerly, it has been confirmed that the semen processing technique can either increase or not alter the sperm chromatin stability. In the present study, the sperm DNA damage is being debated and suggested to be entered as routine semen analysis test; we made an attempt here to evaluate and compare the nuclear integrity of recovered sperm in swim-up and upstream methods. Although the time used in the swim up procedure was longer than the upstream method but this might allow more motile sperm to accumulate. While the sperm processing by the upstream method had a lower motility rate than the swim up method, it could still extract highly motile sperm from the raw semen. 

## Conclusion

This study introduces the better method for separating high qualified sperm. It was shown the upstream method didn’t have any priority on swim up method. In addition, the swim up and upstream methods both had equal ability to select motile sperm with intact DNA compared to raw semen. It should be mentioned that swim up method is still a simple, inexpensive, reliable and widely available method with an efficient yield to separate motile sperm with good morphology and better chromatin integrity for infertility clinics.
